# Lactylation: Metabolic epigenetic regulation and cell death mechanisms in myocardial ischemia‒reperfusion injury

**DOI:** 10.1016/j.gendis.2026.102211

**Published:** 2026-04-22

**Authors:** Xiaoting Yang, Hui Wu, Di Liu, Gang Zhou, Dong Zhang, Yanfang Liu, Yi Li, Tian Zhou, Yan Xiong

**Affiliations:** a Department of Cardiology, The First College of Clinical Medical Science, China Three Gorges University & Yichang Central People's Hospital, Yichang, Hubei 443000, China; b Hubei Key Laboratory of Ischemic Cardiovascular Disease, Yichang, Hubei 443000, China; c Hubei Provincial Clinical Research Center for Ischemic Cardiovascular Disease, Yichang, Hubei 443000, China; d Central Laboratory, The First College of Clinical Medical Science, China Three Gorges University & Yichang Central People's Hospital, Yichang, Hubei 443000, China; e Department of Cardiology, Huangshi Central Hospital, Affiliated Hospital of Hubei Polytechnic University, Huangshi, Hubei 435000, China

**Keywords:** Apoptosis, Autophagy, Ferroptosis, Lactate metabolism, Lactation, Myocardial ischemia/reperfusion injury, Posttranslational modifications, Pyroptosis

## Abstract

One important pathogenic process that contributes to subsequent damage after myocardial infarction (MI) revascularization is myocardial ischemia/reperfusion injury (MI/RI). Its underlying mechanisms are controlled by traditional post-translational modifications (PTMs) like acetylation, methylation, phosphorylation, and ubiquitination, and involve several cell death pathways, such as autophagy, apoptosis, pyroptosis, and ferroptosis. Histone lysine lactylation (Kla), a novel metabolically related epigenetic regulation mechanism, has attracted a lot of attention in recent years. This review systematically clarifies the crucial role of lactate in cardiac energy metabolism and its influence on Kla modification, exploring Kla's dual regulatory function in MI/RI. It demonstrates a significant interaction between Kla and changes like acetylation, phosphorylation, N6-methyladenosine (m6A) methylation, and ubiquitination, thereby establishing putative connections that regulate cell death in MI/RI. Targeting lactate-related molecules such as lactate dehydrogenase A (LDHA), monocarboxylate transporters (MCTs), and histone deacetylases (HDACs) shows therapeutic potential. Therefore, targeted intervention molecules applicable at different stages of MI/RI are explored. Ultimately, elucidating the dynamic patterns of Kla and its crucial involvement in various biological processes will create new paradigms and potential therapeutic targets for the prevention and treatment of MI/RI.

## Introduction

The overall burden of ischemic heart disease (IHD) continues to increase, making it a serious global public health concern.^1^ Numerous processes are linked to the pathological process of ischemia/reperfusion (I/R) injury, which can result in a wide range of cell death and organ damage. Restricting the blood supply to tissues causes ischemia, which results in extreme hypoxia. The subsequent restoration of blood flow, known as myocardial ischemia/reperfusion injury (MI/RI), can, ironically, worsen tissue damage.^2^ Numerous studies show that MI/RI entails a complex interaction of pathological and physiological changes, such as calcium overload, mitochondrial dysfunction, apoptosis, pyroptosis, ferroptosis, and autophagy, all of which lead to oxidative stress and inflammatory reactions.^3^, ^4^, ^5^ Despite significant theoretical advancements in MI/RI treatment over recent decades, challenges persist regarding the clinical applicability, safety, and efficacy of these therapeutic strategies. Therefore, further research into preventive and therapeutic approaches for MI/RI remains of paramount clinical importance.

Lactate is the primary end product of glycolysis and was once erroneously regarded as a mere waste product.^6^ With a deeper understanding of its roles, lactate is now recognized as a key messenger for delivering oxidative and gluconeogenic substrates as well as cellular signals.^7^ Notably, lactate can be produced even under fully aerobic conditions and is utilized by various cell types for multiple functions, serving as a significant energy source, a primary precursor for gluconeogenesis, and a signaling molecule.^8^ Recent studies have revealed that lactate mediates a novel PTM known as lysine Kla, thereby opening new avenues for investigating its physiopathological functions.^8^ Histone lysine lactylation (Kla) is an essential regulatory system in numerous organisms that influences the progression and development of various diseases, including MI/RI.^9^, ^10^, ^11^ In addition, the recently put-out idea of the lactate clock sheds light on the processes controlling the start and stop of Kla.^12^ Lactate is the key factor initiating histone Kla. Intracellular lactate combines with coenzyme A (CoA) to form lactyl-CoA, which subsequently binds to the N-terminal lysine residue of histones, resulting in histone Kla. This modification typically occurs under hypoxic conditions or in response to other stressors.^13^ Intracellular lactate levels directly influence the extent of histone Kla, suggesting that regulating intracellular lactate levels may modulate histone Kla. Lactate exists in two isomers within cells: l-lactate [with an (S) conformation] and d-lactate [with an (R) conformation]. Several studies indicate that histone Kla primarily involves l-lactate rather than d-lactate.^14^^,^^15^ Low intracellular concentrations of d-lactate (ranging from 11 to 70 nM) may contribute to this preference, whereas l-lactate can reach concentrations as high as 40 mM in certain cancer cells.^14^ Intracellular lactate concentrations are influenced by multiple factors, including cell type, metabolic state, oxygen availability, and other environmental factors.^16^, ^17^, ^18^ For instance, blood lactate concentrations may transiently rise to 15 mmol/L during human exercise, whereas resting levels typically range between 1 and 3 mmol/L. During embryonic development, each blastocyst consumes approximately 50–320 pmol of glucose, with approximately 90% converted into lactate.^19^ Lactate is the metabolite with the highest turnover rate in circulation. Its turnover rate is 1.1 times that of glucose during feeding in mice and reaches 2.5 times during fasting, a finding validated through intravenous infusion experiments using ^13^C-labeled tracers.^20^

### Metabolism of lactate in the heart

**Figure 1:**
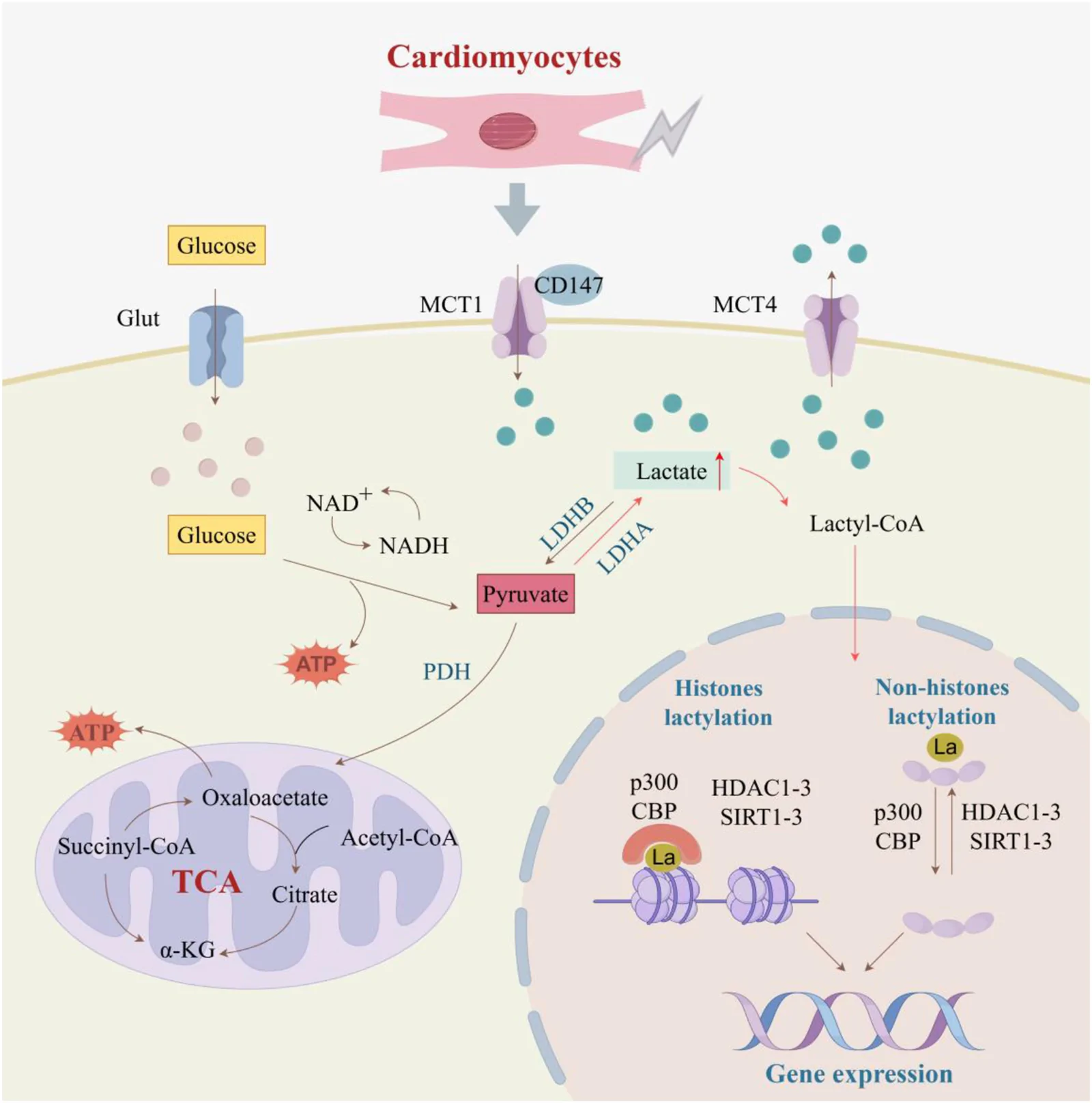
Schematic overview of lactate metabolism in the heart. In response to ischemia, hypoxia, and metabolic disorders, lactate production increases in cardiomyocytes and enters the cells through MCT1. Glucose enters the cytoplasm via glucose transporters (Glut) and is metabolized into pyruvate. Under normoxic conditions, pyruvate is transported into mitochondria, where pyruvate dehydrogenase (PDH) converts it into acetyl-CoA, which then enters the tricarboxylic acid (TCA) cycle for energy production. However, under hypoxic conditions, lactate dehydrogenase A (LDHA) facilitates the conversion of pyruvate into lactate. Furthermore, lactate serves as an important substrate for lysine Kla, a modification that affects both histone and non-histone proteins, ultimately regulating various cellular processes.

Lactate is a vital energy source for organs such as the heart, brain and muscles.^21^, ^22^, ^23^ Cardiomyocytes utilize a variety of substances to meet their energy demands, including lactate, amino acids, and ketone bodies, among others.^24^ Among these, lactate is preferentially metabolized, contributing approximately 10% of the total energy supply under normal physiological conditions. Notably, during exercise, lactate utilization increases significantly, providing over 50% of the energy required by cardiomyocytes.^25^ Within cardiomyocytes, lactate is converted into pyruvate by lactate dehydrogenase (LDH), which then enters the mitochondria and fuels ATP production through the tricarboxylic acid (TCA) cycle, supporting myocardial contraction. Additionally, this metabolic process plays a key role in maintaining systemic acid‒base balance.^26^^,^^27^ In contrast, in the cytoplasm, LDH can catalyze the reverse reaction, converting pyruvate back into lactate^28^(Fig. 1). Clinical studies have shown that myocardial lactate uptake and release increase significantly with elevated cardiac workload, such as during atrial pacing, with lactate release becoming more pronounced as cardiac stress intensifies.^25^^,^^29^ While lactate metabolism helps sustain basal cardiac function under hypoxic conditions, it is not the most efficient energy production pathway.^30^ During MI/RI, intracellular acidification can impair contractile function and exacerbate reperfusion damage.^31^ Interestingly, lactate has been found to promote the maturation and regeneration of neonatal mouse cardiomyocytes and human pluripotent stem cell (hPSC)-derived cardiomyocytes by upregulating BMP10, LIN28, and TCIM while downregulating GRIK1, DGKK, and specific ion channels.^32^ Furthermore, both lactate and pyruvate exhibit cardioprotective effects by scavenging free radicals and mitigating oxidative stress.^33^^,^^34^ Furthermore, lactate influences the electrophysiological activity of cardiomyocytes. For example, it has been shown to activate ATP-sensitive potassium (K ATP) channels in guinea pig ventricular myocytes, thereby shortening the action potential duration.^35^ Several potential mechanisms have been proposed to explain lactate-induced K ATP channel activation: (i) lactate may interfere with ATP production by inhibiting glycolysis; (ii) similar to ADP, lactate may reduce the sensitivity of K ATP channels to ATP^36^^,^^37^; and (iii) lactate may act as a direct channel opener, akin to pharmacological agents such as cromakalim and pinacidil.^38^^,^^39^ However, a recent study reported that lactate can also prolong action potential repolarization by altering the redox state of intracellular nucleotides.^9^ These findings suggest that lactate plays diverse and complex roles in action potential regulation, warranting further research to elucidate the underlying mechanisms.

The bidirectional transfer of lactate between the intracellular and extracellular environments is mediated by a family of transmembrane proteins called monocarboxylate transporters (MCTs), which are located in the lipid bilayer.^40^ There are currently 14 known MCT isoforms.^41^ Among these transporters, MCT1 plays a key role in lactate uptake in cardiomyocytes and is widely expressed across various tissues, including the heart, skeletal muscle, gastrointestinal tract, and kidneys. As a chaperone protein, the monotransmembrane glycoprotein CD147 is necessary for MCT1's location to the cell membrane.^41^^,^^42^ Unlike MCT1, MCT2, MCT3, and MCT4 show tissue-specific expression pattern. MCT4 mainly promotes lactate outflow and has a decreased affinity for lactate. It plays a vital role in lactate clearance and the avoidance of intracellular acidosis in cells with increased glycolytic activity, where it is significantly expressed.^43^^,^^44^ Interestingly, cardiac damage causes a marked upregulation of MCT4 expression in cardiomyocytes, indicating that it plays a role in lactate metabolism reprogramming in pathological conditions.^45^ Studies have demonstrated that MCT1 and MCT4 work synergistically to regulate lactate transmembrane transport.^46^

Under pathological conditions such as acute MI and congenital heart disease, insufficient myocardial perfusion or reduced blood oxygen levels can trigger metabolic reprogramming.^47^ In response, compensatory upregulation of glycolysis leads to increased lactate production, while impaired blood flow hampers lactate clearance, resulting in intracellular lactate accumulation.^48^ Excessive lactate disrupts cardiac function by disturbing ion homeostasis, interfering with contractile protein activity, and inducing electrophysiological abnormalities.^49^^,^^50^ Lactate and its associated Kla changes play a dual role in MI. Kla can have adverse effects; for example, Kla of the transcription factor Snail1 has been shown to facilitate the endothelial–mesenchymal transition (EndoMT), thereby worsening cardiac fibrosis.^51^ Conversely, research suggests that Kla has considerable cardioprotective potential. Studies indicate that the distant activation of early repair genes after MI improves histone Kla. Histone Kla enhances the transcription of the repair genes *Lrg1*, *Vegf-a*, and *IL-10*, thereby cultivating a reparative milieu through their anti-inflammatory and pro-angiogenic functions. Additionally, in a murine MI model, increased histone Kla mitigated detrimental inflammatory responses and enhanced heart function following infarction.^52^ Clarifying the interacting mechanisms between lactate metabolic imbalance and cardiac pathogenic processes is crucial for formulating innovative therapeutic options.

Chronic IHD ultimately results in compromised ventricular function and compliance, rendering cardiac output inadequate to satisfy energy and nutritional requirements, thereby advancing to heart failure (HF). Kla serves a dual role in the progression of HF. Clinical trials suggest that lactate or the lactate-to-albumin ratio (L/A) may function as prognostic indicators for HF post-MI, with heightened lactate levels at admission associated with enhanced mortality rates.^53^^,^^54^ Moreover, HF is often associated with substantial myocardial fibrosis, wherein fibroblasts assume a central role. Studies suggest that increased Kla in fibroblasts may facilitate the fibrotic process, worsening cardiac fibrosis in HF.^55^ Conversely, another study revealed that in HF, lactate directly influences cardiac function via Kla, the essential contractile protein α-myosin heavy chain (α-MHC). Kla at the K1897 site of α-MHC is facilitated by p300 and deacetylated by Sirtuin (SIRT)1; this change enhances the stability of the α-MHC-myosin interaction. In HF, decreased intracellular lactate concentrations hinder this alteration, compromising sarcomere architecture and diminishing cardiac contractility; in contrast, increased lactate levels facilitate this modification and boost cardiac performance.^56^ This counterintuitive occurrence prompts additional inquiries concerning the mechanism of lactate alteration. First, notwithstanding increased plasma lactate concentrations, cardiomyocytes may enhance MCT4 expression to sustain glycolysis in hypoxic environments and eliminate excess lactate, resulting in intracellular lactate deficit and compromised α-MHC Kla. Moreover, the dysregulation of enzymes that regulate Kla may significantly contribute to this phenomenon. P300, functioning as an acetyltransferase, facilitates α-MHC Kla, while SIRT1 operates as a deacetyltransferase that inversely modulates this process. In HF, the dysregulation of p300 and SIRT1 activity may lead to imbalanced Kla levels, undermining the structural integrity and function of cardiac cells, and thus worsening the situation.^57^ Many unsolved inquiries remain in this domain, especially about the opposing mechanisms by which SIRT1 and p300 modulate Kla. Moreover, clarifying the mechanisms by which precise regulation of Kla occurs in various cell types—particularly cardiomyocytes and fibroblasts—will represent critical avenues for future investigation.

### Kla

In eukaryotic cells, nucleoproteins involved in DNA replication, chromosome structure and function maintenance, and gene expression regulation are categorized into histones and non-histones. These proteins undergo various PTMs at specific amino acid residues, including the addition or removal of modifying groups such as acetyl, methyl, or phosphate groups. These modifications profoundly influence epigenetic processes, including chromatin remodeling and gene expression regulation.^58^ The Kla modification discovered in recent years exists in two distinct forms: enzymatic Kla (L-Kla) and non-enzymatic Kla (D-Kla).^8^ These two forms differ in substrate sources, optical activity, target proteins, and functional phenotypes.^8^ Understanding the mechanisms of action for both L-Kla and D-Kla is crucial for elucidating their roles in the pathogenesis of MI/RI. Recent findings have revealed that Kla modification extends beyond histones to encompass non-histone lysine residues. The non-histone Kla interacts with histone Kla, thereby affecting gene expression. For instance, the Kla of transcription factors and DNA repair proteins significantly impacts histone Kla-mediated gene expression.^59^^,^^60^ Moreover, studies have reported that the expression of certain proteins is simultaneously regulated by both histone and non-histone K-lysine modifications, such as neuron-expressed developmental downregulation 4 (NEDD4) and PD-L1.^61^^,^^62^ Consequently, future research should focus on broader epigenetic regulatory networks linking the relationships between histone and non-histone Kla.

### Histone Kla

Histones, as essential structural components of eukaryotic chromatin, associate with DNA to create nucleosomes and are pivotal in preserving chromosomal architecture and functionality.^63^ These proteins undergo various modifications, such as acetylation, phosphorylation, methylation, and sulfidation. These alterations change the spatial structure of proteins, thereby controlling several cellular physiological and biochemical processes.^64^ In 2019, Zhang et al initially identified histone Kla as a new alteration, demonstrating its capacity to enhance chromatin gene expression.^9^ Histone Kla mostly manifests on the lysine residues of H2A, H2B, H3, and H4, especially H3K18.^65^ The crucial function of these sites in disease development has been thoroughly substantiated using various techniques.^9^^,^^66^^,^^67^ As a sign of active gene expression, H3K18la is notably abundant in the promoter and enhancer regions of genes. Furthermore, it is closely linked to a number of physiological and pathological processes, including tumorigenesis, macrophage polarization, stem cell and embryonic development, and gene transcriptional control.^65^ Several additional histone Kla sites, such as H2BK6, H3K9, H3K14, H3K23, H3K56, H4K5, H4K80, and H4K12, have been found to play regulatory roles in addition to H3K18.^68^ Research has indicated that H3K9la may increase the transcription of genes in important signaling pathways, including Wnt, Hippo, MAPK, and PI3K-AKT.^69^ Additionally, higher levels of H4K12la were found on the promoters of NF-κB pathway genes, including Ikbkb, Rela, and Relb, in an animal model of I/R.^70^ Kla may affect how proteins function in two main ways: (i) Kla can directly alter non-histone proteins, controlling their biological function; (ii) Kla can directly attach to promoter areas, modifying gene expression. According to recent research, Kla is extensively found on non-histone lysine residues and is not confined toon histones. Gene expression is further influenced by the interaction between histone and non-histone Kla. For example, it has been demonstrated that histone Kla-mediated gene regulation is greatly impacted by the Kla of transcription factors and DNA repair proteins.^59^^,^^60^

### Non-histone Kla

The non-histone Kla process refers to the process in which the lysyl groups on proteins (rather than histones) combine with lysine residues.^71^ Unlike histone Kla, which mainly affects gene transcription and chromatin structure, non-histone Kla has a more extensive role, directly influencing protein function and thereby participating in the regulation of various biological processes, such as cell proliferation, differentiation, and programmed cell death, and is of great significance in the body's responses to stress reactions such as inflammation and tissue repair.^72^

Non-histone Kla plays a significant role in metabolic control, signal transmission, and immune responses. The study by Yang et al.^73^ offers significant insights into the extensive role of non-histone Kla in regulating metabolic processes, signaling pathways, and immunological responses. This underscores its significance in several diseases, including cancer, metabolic syndrome, and neurodegenerative disorders. The finding of unique Kla sites on essential metabolic enzymes and regulatory proteins, including adenylate kinase 2 (AK2) and aldolase A (ALDOA), in hepatocellular carcinoma highlights the intricacy of Kla networks and their influence on cellular and metabolic activities.^74^ In metabolic diseases, particular Kla locations on fatty acid synthase (FASN) in MPC1^+/−^ knockout mice signify impaired fatty acid production and metabolism, associated with essential mitochondrial function and metabolic reprogramming.^75^ With the development of mass spectrometry techniques and deep sequencing methods, researchers have identified a large number of lactate-producing non-histone proteins (such as high-mobility group box protein 1(HMGB1), methyltransferase-like 3(METTL3), pyruvate kinase muscle isozyme M2(PKM2), and AK2) in various disease models, including sepsis, liver ischemia-reperfusion injury, colon cancer, and hepatocellular carcinoma.^74^^,^^76^, ^77^, ^78^ The findings indicate that non-histone Kla, a crucial post-translational modification pathway, may provide new therapeutic targets for a range of diseases, including cardiovascular ailments. Future investigations should clarify the precise role of non-histone Kla in MI/RI, including its involvement in cardiac metabolic regulation and stress responses. This will furnish essential theoretical underpinnings for the formulation of innovative therapeutic approaches.

## Regulation of Kla modification

### Enzymatic Kla

Histone Kla is a reversible dynamic modification whose homeostasis is sustained by the coordinated activity of three functional molecule groups: “writers” that promote the attachment of lactyl groups to target proteins (encompassing both histone and non-histone proteins), “erasers” that remove these lactyl groups, thus allowing for dynamic and reversible regulation of Kla modification, and effector “readers”.^79^ These identify the changes and convert them into various cellular functions by regulating downstream signaling pathways. This complicated system of enzymes and proteins facilitates the precise regulation of Kla, highlighting its significance as a crucial epigenetic regulator.^80^^,^^81^ As of now, the exact readers for Kla have not been identified. Current study posits that chromatin modification factors, including bromodomains, may be involved in the recognition of lactated histones; however, this mechanism necessitates additional validation.^82^

Zhang et al first documented the lactyl transferase activity of p300 in 2019, suggesting that L-lactyl-CoA functions as a substrate for histone Kla in an enzymatic context, with a dose-dependent correlation identified between intracellular lactate concentrations and L-Kla.^9^ The structure of the multifunctional protein p300 and its homolog cAMP-response element-binding protein (CBP) consists of a catalytic domain that functions as an acetyl-lysine “writer” and a bromodomain (BRD) that functions as an acetyl-lysine “reader” to identify and bind acetylated proteins.^83^ Subsequent investigations validated that p300 and its homolog cAMP-response element-binding protein (CBP) promote the Kla of HMGB1, underscoring the potential function of the p300/CBP complex in modulating histone Kla.^76^ It has also been discovered that other lysine acetyltransferases possess lactyl transferase activity. The HBO1 protein, for example, has a negatively regulated N-terminal region and a catalytically active C-terminal MYST domain that promotes gene transcription by catalyzing the Kla of both histone H3K9.^9^^,^^69^ Recent studies further indicate that KAT5 and KAT8 can also regulate the lactylation levels of non-histones.^61^^,^^84^ The C-terminus of KAT5 comprises a MYST-type HAT domain, while its N-terminus contains a Tudor knot domain^85^; KAT8 possesses an N-terminal chromatin domain and a centrally located MYST histone acetyltransferase domain.^86^ In addition to the traditional acetyltransferase p300/CBP, which has demonstrated lactyl transferase activity, non-canonical Kla pathways have surfaced in recent years. Zong et al hypothesized an alternate mechanism in which lactyl groups are directly transferred to lysine residues through alanine-tRNA synthetase (AARS1), resulting in lysyl Kla.^87^ As a member of the second class of the AARS family, AARS1/2 has an antiparallel β-fold catalytic domain, as well as tRNA-binding, editing, and C-terminal domains. The C-terminal domain of AARS1 contains a nuclear localization motif, while the AARS2 protein is located within mitochondria.^88^ Subsequent research demonstrated that AARS1/2 function as global lysine lactyl transferases, directly employing lactyl-AMP to facilitate the ATP-dependent transamination of lysine residues at Kla site.^89^

HDAC1–3 and SIRT1–3 are recognized as prominent “de-modifiers”. HDAC1–3 demonstrate deacetylase activity. Their catalytic domains are highly conserved, with a core β-sheet surrounded by helices and disordered loops. The zinc ion necessary for catalysis is firmly implanted in the base of a tubular channel that is approximately 11 Å long, and the active site is located at the opening on the enzyme's surface. The “foot pocket,” a channel approximately 14 Å long that intersects this channel perpendicularly, is in charge of discharging the acetic acid product produced during the deacetylation reaction.^90^^,^^91^ Researchers conducted a groundbreaking study that comprehensively examined all 18 HDACs. Class I HDACs, especially HDAC1–3, were identified as the most effective 'erasers' of Kla alterations *in vitro*.^9^ Notably, HDAC1 and HDAC3 demonstrate substantial de-Kla activity in vivo, thus reinforcing their role as the principal 'erasers' of Kla. Furthermore, broad-spectrum HDAC inhibitors such as sodium butyrate and trichostatin A (TSA) efficiently eliminate histone Kla modifications. These inhibitors usually work by occupying the substrate-binding channel in the HDAC active site or chelating zinc ions, demonstrating how knowledge of enzyme structure serves as a foundation for medication intervention. This discovery further substantiates the pivotal function of HDAC1–3 in Kla processes.^9^^,^^14^ In contrast, SIRT1 and SIRT3 effectively eliminate Kla from both histone and non-histone proteins.^14^^,^^19^ They consist of a minor domain with a helical and zinc-binding module and a major, highly conserved Rossmann-fold domain. The target molecule's acetylated residue binds to NAD ^+^ across the gap between these two domains to start catalytic events.^92^ SIRT1 is involved in physiological processes intimately associated with cardiovascular health, such as the regulation of vasodilation and vasoconstriction, mitigation of oxidative stress, inhibition of inflammatory responses, modulation of metabolic processes and protection of cardiomyocytes.^93^ Recent investigations have elucidated SIRT1's function in regulating cardiac Kla by serving as a de-lactylating enzyme for α-MHC K1897, therefore affecting its Kla levels.

### Non-enzymatic Kla

In contrast to the L-Kla process delineated by Zhang et al, Gaffney et al suggested a non-enzymatic mechanism for D-Kla. It was proposed that lactylcysteine (LGSH), a metabolite of methylglyoxal (MGO), may directly lactylate lysine residues. This hypothesis was corroborated by *in vitro* experiments, in which the co-incubation of histone H4 with LGSH markedly elevated lactyllysine levels.^15^ LGSH is produced when glucuronosyltransferase 1 (GLO1), a thioesterase, catalyzes the conjugation of MGO with glutathione. GLO2 subsequently hydrolyzes LGSH into d-lactate and glutathione.^15^ Trugillo et al showed that lactate spontaneously transferred from LGSH to CoA via S-to-S acyl transfer, producing lactoyl-CoA, which in turn causes lysine D-Kla on histones.^94^ These results suggest that through Kla, lactate and LGSH both contribute to intracellular alterations. Notably, lactate-induced Kla and LGSH-associated Kla have different biological effects: lactate-induced Kla encourages macrophage polarization toward an anti-inflammatory phenotype, while LGSH-associated Kla is linked to elevated pro-inflammatory cytokine production in macrophages.^9^^,^^95^ Further studies are required to elucidate the intricate regulatory networks governing lactate-induced and LGSH-associated Kla in cellular processes.

### Crosstalk between Kla and PTMs

PTMs of proteins play a crucial role in living organisms, altering the properties and functions of proteins.^96^ Common PTMs include methylation, acetylation, phosphorylation, ubiquitination, sumoylation, glycosylation, butylation, succinylation, and propionylation. Kla transpires at the ε-amino group of lysine residues, where it competes with many protein modifications targeting the same site, including acetylation, ubiquitination, methylation, and SUMOylation.^15^ Recent studies suggest that the bulk of PTMs are not found in isolation. Conversely, any two or more unique PTMs may interact, with these combined PTM states possibly augmenting or suppressing one another.^68^ This review examines the relationships between Kla and acetylation, ubiquitination, phosphorylation, and SUMOylation, as the relationship between Kla and other protein modification processes is inadequately researched. We expect forthcoming studies to enhance our comprehension of these connections.

### Kla and acetylation

Kla and acetylation, two essential PTMs of proteins, utilize the same regulatory enzyme systems: both are catalyzed by P300/CBP family proteins serving as “writers” and are removed by class I histone deacetylases, such as HDAC1–3, acting as “erasers”.^9^^,^^14^ This indicates that Kla and acetylation may demonstrate synergistic regulation in cellular biological processes. The predominance of either modification is influenced by various parameters, including substrate concentration (the ratio of acetyl-CoA to lactyl-CoA), differential cofactor recruitment, kinetic features of the modifications, and the specific activity of the modifying enzymes.^11^

In terms of metabolism, both lactyl-CoA and acetyl-CoA originate from pyruvate. Hypoxia facilitates the conversion of pyruvate into lactate and lactyl-CoA, while in oxygen-rich environments, pyruvate is mostly transformed into acetyl-CoA to participate in the TCA cycle.^97^ Kla and acetylation exhibit certain similarities; however, the duration necessary for Kla to attain a steady-state modification level (approximately 24 h) is considerably longer than that required for acetylation (approximately 6 h). This suggests that acetylation possesses a kinetic advantage in physiological settings.^98^

Kla and acetylation functionally interact competitively, producing divergent effects in various clinical diseases. For example, in hepatic fibrosis, lactate facilitates Kla at H3K18 while simultaneously blocking acetylation at the same locus, thereby modifying gene expression patterns, activating hepatic stellate cells and propelling fibrotic development.^99^ In macrophages, lactate concurrently promotes Kla and acetylation of the HMGB1 protein, aiding its transport from the nucleus to the cytoplasm and subsequent exosomal release, thus undermining endothelial integrity.^76^ In pulmonary epithelial cells, AARS1-mediated Kla of the lysophosphatidylcholine acyltransferase 2 (LPCAT2) protein at position K375 suppresses STAT1 acetylation, boosting its phosphorylation and nuclear translocation, which eventually induces ferroptosis.^100^ TEC-mediated tRF-31R9J inhibits hepatic ferroptosis by HDAC1-regulated histone deacetylation, therefore alleviating non-alcoholic steatohepatitis (NASH).^101^

### Kla and methylation

Lactate modulates m6A regulation through both histone and non-histone Kla. Studies have shown that elevated lactate levels induce histone Kla and regulate m6A-RRE activity, thereby influencing m6A modifications in downstream targets associated with disease progression.^102^^,^^103^ Lactate-induced Kla at H3K18 upregulates METTL3 in tumor-infiltrating myeloid cells, which then alters the oncogene Janus kinase 1 (JAK1) via m6A, enabling tumor immune evasion.^77^ It is noteworthy that H3K18la is abundant in the METTL3 promoter region, increasing its transcription, whereas p300 enzyme inhibition lowers METTL3 and Kla levels.^77^ In macrophages, H3K18la enhances the expression of anti-inflammatory genes, potentially mitigating the repressive effects of methylation at neighboring loci.^65^ Lactate, serving as a substrate for Kla, indirectly impacts methylation by modifying S-adenosylmethionine (SAM) availability via factors like hypoxia-inducible factors (*e.g.*, HIF-1α), therefore influencing the Kla process.^104^ Similarly, the lactate-mediated H3K18la in the FTO promoter region stimulates its expression by a similar mechanism.^103^ To improve the detection and degradation of m6A-modified cyclic circadian protein homolog 1 (PER1) and tumor protein 53 (TP53) mRNA and speed up tumor progression, Kla of H3K18 directly controlls the expression of YTHDF2.^102^ These findings suggest that H3K18 is a crucial site for lactate-induced histone Kla in the regulation of m6A modification. This mechanism predominantly enhances the transcriptional regulation of m6A-RRE targets, affecting multiple biological processes. Nevertheless, the exact mechanisms underlying this regulation remain to be fully explored. Notably, lactate also plays a role in modulating non-histone protein Kla, which can impact protein function. For instance, Kla of ALKBH5 enhances its binding to interferon-β (IFN-β) mRNA, promoting the demethylation of m6A and consequently boosting IFN-β biosynthesis.^105^ Furthermore, Kla of METTL16 facilitates methylation at the FDX1 602 site, which in turn upregulates FDX1 expression and triggers copper-dependent cell death.^106^ These findings indicate that non-histone Kla has the potential to regulate both protein expression and function, although the precise regulatory pathways involved require further clarification.

### Kla and phosphorylation

Numerous studies have explored the interactions between phosphorylation and Kla, which often exhibit synergistic effects. For instance, Maschari et al^107^ found that protein Kla and IRS-1 serine 636 phosphorylation are induced in a dose-dependent manner during exposure to sodium lactate. Similarly, Xu et al^108^ showed that Sox10 Kla is induced by tumor necrosis factor-alpha (TNF-α) via a phosphorylation-dependent mechanism within the PI3K/AKT pathway. Additionally, Wang et al^109^ found that by altering the phosphorylation of epidermal growth factor receptor (EGFR), which in turn affects the mitogen-activated protein kinase (MAPK) signaling pathway, methylated CpG binding protein 2 (MeCP2) K271 Kla reduces atherosclerosis and suppresses the expression of epithelial regulatory proteins. Additionally, during *Staphylococcus aureus* infection, methylthiomethane stimulates STAT3 phosphorylation and increases the expression of target genes specific to H3K18la.^110^ Xiong et al^77^ further demonstrated that lactate upregulates METTL3, activates downstream signaling, and enhances STAT3 phosphorylation via H3K18la**.** Through its C-terminal region, FK506-binding protein 10 (FKBP10) interacts with LDHA in renal clear cell carcinoma, promoting the phosphorylation of LDHA tyrosine 10 (LDHA-Y10). Histone Kla accumulation is caused by the hyperactive Warburg effect, which is fueled by this interaction.^62^ On the other hand, Kla alteration of AMPKα promotes cellular senescence and autophagy by inhibiting its phosphorylation.^111^ These results imply that the structural characteristics of particular substrate active sites may be responsible for the varied control of Kla and phosphorylation. Overall, there are several levels at which phosphorylation and Kla interact, and understanding the processes driving this connection is essential to understanding how cellular metabolic and signaling pathways are coupled.

### Kla and ubiquitination

Ubiquitination is a prevalent protein modification that governs protein stability through the ubiquitin‒proteasome system, significantly influencing cell death. Recent studies demonstrate that Kla regulates cell death by altering the expression of ubiquitin ligases and directly influencing their activity via competitive inhibition.^112^ Ubiquitination also occurs on lysine residues and competes directly with Kla, affecting the stability of proteins. Studies have demonstrated that lactate stimulates the production of the deubiquitinating enzyme proteasome 26S subunit, non-ATPase 14 (PSMD14), by increasing histone Kla levels. This eliminates the ubiquitin modification of PKM, thereby enhancing mitochondrial autophagy to diminish reactive oxygen species (ROS).^113^ Kla at K91 of transcription factor EB (TFEB) obstructs its interaction with the E3 proteasome ligase WW domain-containing protein 2 (WWP2), thereby preventing TFEB ubiquitination and proteasomal degradation. This substantially improves cellular autophagy activity and lysosomal function.^60^ Increased lactate concentrations induce PCBP2K115la alteration, impairing PCBP2's association with ARIH2, thereby obstructing ubiquitin-mediated degradation and enhancing PCBP2 stability.^114^ In aortic aneurysms and dissections, the E3 ubiquitin ligase March 2 facilitates the transformation of PKM2 dimers into tetramers via polyubiquitination at the K33 site, consequently suppressing lactate generation. Downregulation of March 2 results in lactate build-up, which increases histone H3K18la and induces apoptosis by activating the p53 transcriptional pathway.^115^ Furthermore, lactate directly facilitates the Kla of NEDD4 by attaching lactyl groups to proteins. This mechanism obstructs NEDD4's association with Caspase-11, consequently diminishing Caspase-11 ubiquitination and increasing its protein concentrations, thereby intensifying non-canonical pyroptosis.^116^ Recent studies indicate that Kla directly facilitates ubiquitin-independent proteasomal degradation of proteins, offering a new paradigm for the regulation of protein stability. Kla at the K21 region of the cGAS protein directly facilitates its interaction with the proteasome subunit PSMA4, resulting in degradation that completely circumvents the conventional ubiquitination pathway.^117^ This indicates that Kla is not only a competing alteration to ubiquitination but may represent a distinct protein degradation process.

### The regulation of Kla in MI/RI

In recent years, the significance of Kla in MI/RI has been progressively clarified; however, the fundamental processes governing its functional transformation remain ambiguous. Current research suggests that Kla may have preventive effects while also intensifying damage in MI/RI. A summary of histone and non-histone Kla and dual regulatory molecules in MI/RI is presented in Table 1.

**Table 1:** Histone Kla and -histone Kla in MI/RI, as well as the dual role of Kla

Lactylation	Target	Modification trend	Target gene expression and function	Regulatory mechanism	Effect	References
Histone	H3K56	Lactylation ↑	Not determined	Improving cardiomyocyte survival	Protection	118
	H3K18	Lactylation ↑	Promote the transcription and protein stability of MICU3	Activate mitotophagy	Damage	119
	H3K18	Lactylation ↑	Promote the transcription of TRPM7	Promote apoptosis	Damage	120
	H3K18	Lactylation↑	Promote the expression of YTHDF2 protein	Promote apoptosis	Damage	121
Non-histone	Serpina3k k351	Lactylation↑	Inhibits the WNT pathway and activates RISK and SAFE pathways	Inhibition of apoptosis	Protection	127
	S100a9 K26	Lactylation↑	Promote the mRNA expression of Itga2, Rhoj and Cxcr1	Leading to cardiomyocyte death by disrupting mitochondrial function	Damage	122
	GPX4 K218 and K228	Lactylation↑	Reduce the stability of the GPX4 protein	Promote ferroptosis	Damage	123
	ACSL4 K83	Lactylation↑	Enhance the stability of ASCL4 protein	Promote ferroptosis	Damage	124
	MDH2 K241	Lactylation↑	Not determined	Induce mitochondrial dysfunction and promote ferroptosis	Damage	126
	NLRP3 K245	Lactylation↑	Enhance the stability of NLRP3 protein	Promote pyroptosis	Damage	125

### Histone Kla in MI/RI

Recently, the mechanism of lactate-induced Kla in MI/RI has attracted considerable interest. Yu et al^118^ noted that heat shock protein 12A (HSPA12A) stabilizes hypoxia-inducible factor-1α (HIF-1α) protein through SMAD-specific E3 ubiquitin protein ligase 1 (SMURF1), consequently augmenting glycolytic gene expression, preserving aerobic glycolysis, sustaining H3K56la, and promoting cardiomyocyte lifespan, thereby alleviating MI/RI. Lactate can improve the transcriptional process and protein stability of MICU3 through H3K18la, ultimately activating neutrophil extracellular traps (NETs), which leads to microvascular endothelial cell injury and exacerbates MI/RI.^119^ MI/RI enhances the transcription of the calcium channel protein TRPM7 by H3K18la activation; the overexpression of TRPM7 triggers apoptosis in MI/RI, whereas the suppression of TRPM7 significantly reduces apoptosis in MI/RI.^120^ Moreover, exercise intervention studies demonstrate that swimming training confers a protective effect against MI/RI by decreasing lactate levels and histone H3K18la modification in mouse cardiac tissue, consequently downregulating YTHDF2 expression.^121^

### Non-histone Kla in MI/RI

Non-histone Kla demonstrates a notable correlation with MI/RI. Studies demonstrate that the EndoMT is pivotal in the pathological progression of cardiac fibrosis. Lactate specifically promotes the EndoMT after MI, consequently worsening cardiac fibrosis and impairing cardiac function, with the Kla of Snail1 being crucial in this intricate mechanism.^51^ Furthermore, in MI/RI, increased Kla of S100a9 in neutrophils results in a collapse of cellular energy metabolism and subsequent cardiomyocyte death.^122^ In MI/RI, glycolysis enhances the Kla of GPX4 at residues K218 and K228, leading to the destabilization of the GPX4 protein and subsequently promoting ferroptosis in cardiomyocytes.^123^ Kla during MI/RI improves the stability of the ACSL4 protein, consequently facilitating ferroptosis. Knockout of LDHA lowers lactate concentrations and ACSL4 expression, thereby decreasing ferroptosis and alleviating MI/RI.^124^ Additionally, LDHA facilitates pyroptosis-mediated cardiac ischemia/reperfusion damage through the upregulation of NLRP3 Kla.^125^ Dexamethasone (Dex) downregulates PDK4 by enhancing NR3C1 phosphorylation, resulting in diminished lactate generation and lowered MDH2 K241 Kla levels, thereby mitigating MI/RI.^126^ However, studies demonstrate that Kla changes confer protective effects against MI/RI. The Serpina3k protein is lactylated at the K351 location in fibroblasts. Activated fibroblasts produce lactylated Serpina3k extracellularly, exerting paracrine effects on cardiomyocytes. By activating the cardioprotective RISK and SAFE signaling pathways, it significantly suppresses cardiomyocyte apoptosis produced by MI/RI.^127^

### Potential causes contributing to the dual action of Kla

Notably, Kla has been reported to present various, sometimes even opposite, effects in different studies. While the precise mechanism has yet to be fully elucidated, current evidence suggests that the functional switch of Kla may be attributed to cell type-specific Kla profiles, variations in lactate concentrations, and distinct histone Kla enrichment across genes. Following MI, elevated lactate levels can trigger H3K18la in circulating monocytes, inducing the activation of repair genes.^52^ Meanwhile, the acidic environment following MI has been proven to promote the Kla of Snail1 in endothelial cells, leading to the transformation of the EndoMT, thereby having a detrimental effect on cardiac repair.^51^ In MI/RI, elevated lactate levels promote cardioprotection by facilitating Kla of Serpina3k in fibroblasts^127^ but simultaneously induce myocardial inflammation and pathological injury through Kla of S100a9K26 in neutrophils.^122^ Moreover, recent research on the role of Kla in tumors suggests an alternative explanation, indicating that the threshold of lactate concentrations may be significant. It has been found that the antitumor immunity of CD ^8+^ T cells is enhanced with increasing lactate concentrations and subsequent activation of H3K18la.^128^ However, within the tumor microenvironment, the local concentration of lactate is typically influenced by the metabolic patterns of tumor cells and often exceeds a potential threshold (> 10 μM). Beyond this point, the regulatory effect of Kla may shift to facilitate tumor growth by inhibiting the activity of the tumor suppressor p53 through K120 and K139 Kla.^87^ Additionally, the cytotoxic activity of CD^8+^ T cells is disrupted via the MondoA-TXNIP-dependent pathway in an immunosuppressive microenvironment induced by excessively high lactate concentrations.^129^ Histone Kla, a recently discovered epigenetic modification, exhibits significant variability in its enrichment pattern and targeting pathways across different histone lysine residues, which may also cause bidirectional regulatory effects. The enrichment analyses of CD^8+^ T cells indicated that the enrichment of H3K9la peaked in naïve CD^8+^ T cells, predominantly influencing pathways related to oxidative phosphorylation and mitochondrial metabolism. Conversely, the chromatin region enriched with H3K18 was more indicative of CD8^+^ T cells activation, as well as inflammatory responses and glycolytic metabolic pathways.^128^

### Mechanisms by which Kla-PTMs regulate cell death in MI/RI

The pathogenic mechanism of MI/RI is intricately linked to the aberrant activation of programmed cell death. In recent years, lactate, a significant metabolite, and its unique PTM (Kla), have been shown to play a critical regulatory role in MI/RI. Kla directly alters functional proteins and interacts extensively with several PTMs, including phosphorylation, acetylation, methylation, and ubiquitination. Collectively, these constitute a meticulously calibrated regulatory network that significantly impacts the development and results of many cell death pathways, including autophagy, apoptosis, pyroptosis, and ferroptosis. The subsequent sections will thoroughly clarify the intricate regulatory functions of Kla and its PTM synergistic network in cell death during MI/RI.

### The regulation of autophagy by Kla-PTMs in MI/RI

Autophagy is a lysosome-dependent degradation mechanism that allows cells to preserve homeostasis and respond to stress.^130^ This conserved lysosomal degradation process is also controlled by Kla.^131^ Studies have demonstrated that lactation can influence autophagic flux by directly altering fundamental autophagic components. The autophagy-initiating kinase ULK1 phosphorylates the glycolytic enzyme LDHA, thereby augmenting its activity to facilitate lactate generation. This then induces the Kla of the vacuolar sorting protein Vps34. Kla of Vps34 facilitates autophagosome formation and maturation while regulating the endosomal–lysosomal degradation pathway, therefore increasing autophagy activation.^61^ Conversely, Kla also inhibits autophagy. Glutamine diminishes lactate generation by decreasing glycolysis, which downregulates AMPKα Kla while concurrently enhancing its phosphorylation. This ultimately improves cellular autophagy and postpones aging processes. Kla of the mitochondrial protein Tu translation elongation factor, mitochondrial (Tufm), similarly obstructs its interaction with the mitochondrial membrane protein TOMM40, consequently hindering Tufm's mitochondrial localization and exerting an inhibitory influence on mitophagy.^132^

Kla in MI/RI interacts with other PTMs to modulate autophagy. Lactate changes H3K18 by Kla in the mitochondrial calcium uptake family 3 (MICU3) promoter region of neutrophils, thereby augmenting its transcription and increasing MICU3 levels. Moreover, lactate facilitates the interaction between MICU3 and AASR1, hence stabilizing MICU3 via Kla. Enhanced MICU3 interacts with voltage-dependent anion channel 1 (VDAC1), facilitating the activation of mitophagy in MI/RI.^119^ Moreover, MICU3 deletion in MI/RI alleviates myocardial damage and enhances functional recovery by diminishing mitochondrial calcium excess, resulting in increased levels of phosphorylated pyruvate dehydrogenase (PDH).^133^^,^^134^ Consequently, MICU3 may act as a crucial nexus between lactate and phosphorylation changes, governed by lactate concentrations, potentially affecting the phosphorylation state of essential downstream metabolic enzymes by regulating mitochondrial calcium homeostasis. This method elucidates a potential mechanism by which lactate and phosphorylation may collaboratively interact via MICU3 to co-regulate autophagy processes and cellular fate in MI/RI. Additional examination of the precise mechanisms by which these two pathways modulate autophagy in MI/RI necessitates future inquiry.

### The regulation of apoptosis by Kla-PTMs in MI/RI

Apoptosis is likely the most common type of programmed cell death, requiring specialized cysteine-dependent aspartate-specific proteases (caspases) for the systematic breakdown of the cell.^135^ In MI/RI, Kla transpires at the K351 locus of the Serpina3k protein in fibroblasts. Activated fibroblasts exude this lactate-modified Serpina3k extracellularly, where it exerts paracrine effects on cardiomyocytes. By activating the cardioprotective RISK and SAFE signaling pathways, it significantly inhibits reperfusion-induced cardiomyocyte death.^127^ Studies indicate that metabolic reprogramming after MI/RI increases lactate levels, thereby enhancing H3K18la. This initiates the transcription of the calcium channel protein transient receptor potential cation channel member 7 (TRPM7), whose overexpression causes pathological calcium influx, ultimately leading to cardiomyocyte death. Inhibition of TRPM7 significantly reduces cell death in MI/RI.^120^

In MI, the interaction between Kla and other post-translational changes governs apoptosis. Studies demonstrate that astragaloside IV (AS-IV) safeguards cardiomyocytes against apoptosis during MI by regulating the equilibrium between Kla and acetylation changes. The fundamental mechanism involves AS-IV's ability to lower lactate concentrations in M1 macrophages and damaged cardiac tissue, consequently preventing the Kla of the transcription factor Snail1. This mechanism may competitively enhance Snail1 acetylation, ultimately inhibiting the activation of the pro-apoptotic TGF-β signaling pathway.^136^ Kla concurrently enhances apoptosis through non-canonical m6A regulatory mechanisms. During MI/RI, elevated lactate levels increase protein Kla, therby particularly augmenting the production of the m6A reader protein YTHDF2. The increased YTHDF2 enhances the production of GTPase-activating protein-binding protein 1 (G3BP1), thereby intensifying cardiomyocyte apoptosis.^121^ It is worth noting that this study demonstrates that YTHDF2 regulates G3BP1 not through its conventional RNA-binding domain that recognizes m6A sites but instead through its intrinsic disordered region (IDR).^121^ This indicates that Kla may serve as an upstream signal that directly modulates the expression levels of m6A-reading proteins. Subsequent research on Kla and essential m6A enzymes in MI/RI—including the ‘writer enzymes’ METTL3 and METTL14, as well as the ‘eraser enzymes’ FTO and ALKBH5—will greatly enhance our comprehension of Kla-methylation interactions in MI/RI.

### The regulation of pyroptosis by Kla-PTMs in MI/RI

Pyroptosis is a type of programmed cell death distinguished by cellular swelling, membrane rupture, the creation of pore channels in the plasma membrane and the release of pro-inflammatory mediators.^137^ Recent investigations indicate functional relationships between Kla changes and other PTMs, which jointly regulate pyroptosis. Tumor necrosis factor-α (TNF-α) activates the PI3K/AKT signaling pathway through a phosphorylation-dependent mechanism, which subsequently induces Kla modification of the transcription factor Sox10. This induces phenotypic transdifferentiation in vascular smooth muscle cells (VSMCs) and facilitates pyroptosis.^108^ Kla has been identified as a crucial mechanism triggering cardiomyocyte pyroptosis in MI/RI. LDHA specifically facilitates NLRP3 inflammasome activation by Kla, thereby intensifying cardiomyocyte pyroptosis and eventually aggravating I/R injury.^125^ In contrast, reducing LDHA expression markedly diminishes hypoxia/reoxygenation (H/*R*)-induced cardiomyocyte pyroptosis.^125^

Kla may interact with acetylation and ubiquitination. Research demonstrates that KAT5 facilitates the ubiquitination and degradation of large tumor suppressor kinase 2 (LATS2) protein through the acetylation of STUB1, consequently reducing NLRP3-mediated pyroptosis to alleviate MI/RI.^138^ KAT5 has been shown to behave as a ‘writer’ of Kla, regulating non-histone Kla levels.^139^^,^^140^ These findings elucidate the intricate relationships between Kla and other PTMs within the pyroptosis regulatory network, providing novel insights into the molecular mechanisms governing programmed cell death. In summary, throughout the MI/RI process, NLRP3, as a pivotal molecule in pyroptosis, integrates several PTM signals, including Kla, phosphorylation, and acetylation, thereby establishing a complex regulatory network. Future studies must clarify the precise sites of action for these modifications on NLRP3, their interconnections, and their dynamic changes under pathological conditions. This will establish innovative theoretical frameworks and therapeutic targets for the prevention and treatment of MI/RI.

### The regulation of ferroptosis by Kla-PTMs in MI/RI

Ferroptosis is an iron-dependent, non-apoptotic type of programmed cell death induced by lipid peroxidation of polyunsaturated fatty acids in biological membranes.^141^ Kla plays a role in modulating ferroptosis during MI/RI. The activation of glycolysis in cardiomyocytes subjected to I/*R* exacerbates cellular damage. Specifically, glycolysis facilitates the Kla of GPX4 at residues K218 and K228, therefore destabilizing the GPX4 protein. This promotes ferroptosis in cardiomyocytes and exacerbates cardiac damage in the MI/RI rat model.^123^ In H/R-treated cardiomyocytes, the stability of the ACSL4 protein is augmented, while LDHA deletion diminishes ACSL4 expression, lactate concentrations, and stability, indicating that lactate oxidation may bolster ACSL4 protein stability. Moreover, LDHA deletion inhibited ferroptosis in H/R-treated H9C2 cells, an effect negated by ACSL4 overexpression.^124^ Crosstalk between Kla and phosphorylation regulation influences ferroptosis in MI/RI. Dex exemplifies its cardioprotective properties. Research demonstrates that Dex inhibits lactate generation by enhancing the phosphorylation of the glucocorticoid receptor (NR3C1), thereby suppressing the expression of its downstream target PDK4.^126^ The decrease in lactate levels directly reduces Kla at the K241 location of malate dehydrogenase 2 (MDH2), thereby alleviating ferroptosis in MI/RI.^126^ In MI/RI, Kla establishes a synergistic regulatory network with phosphorylation changes, together influencing the course and outcome of ferroptosis by precisely modifying critical proteins. Future research may further explore the regulatory functions of Kla and other PTMs in ferroptosis during MI/RI.

**Figure 2:**
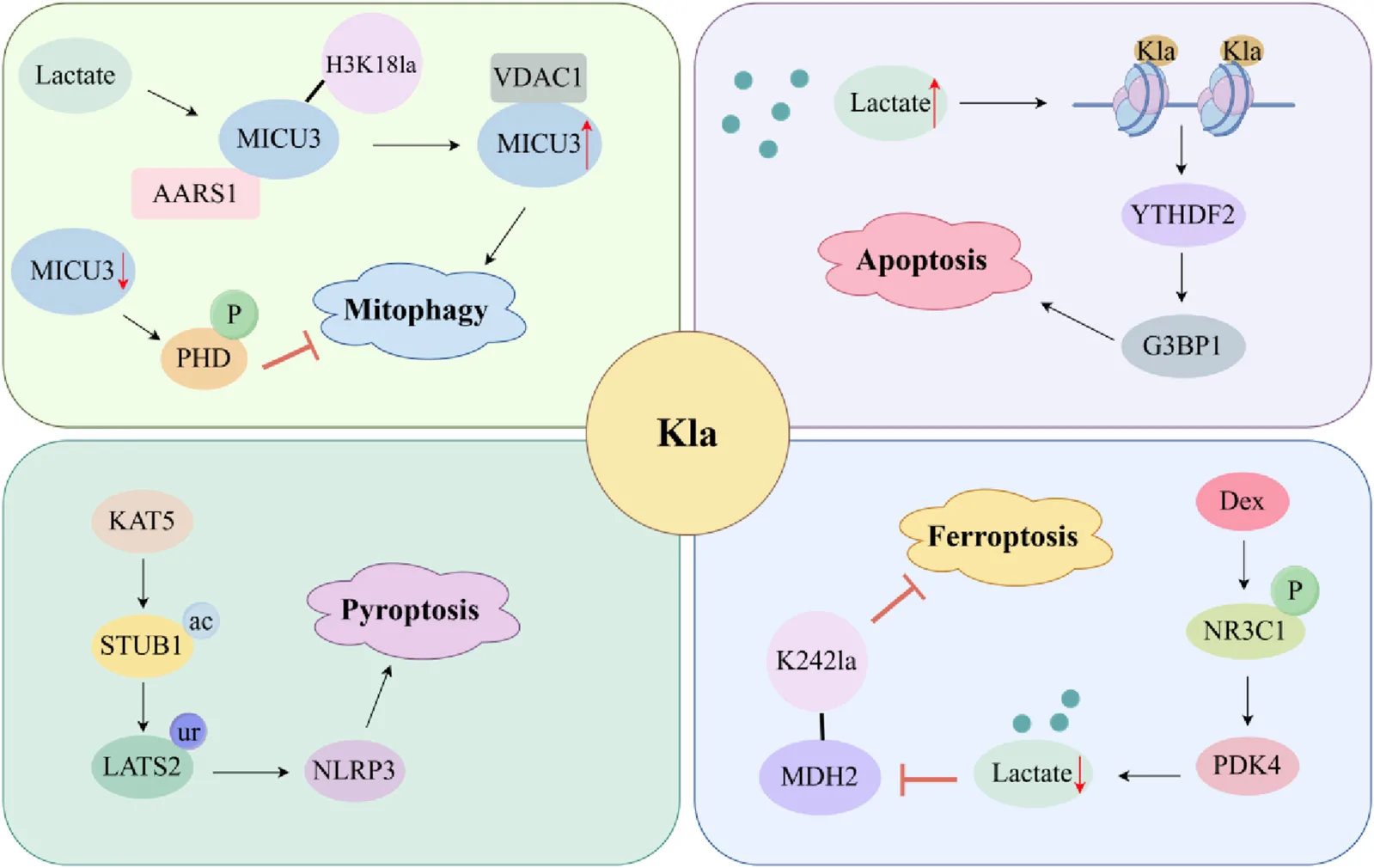
Potential mechanism of histone lysine lactylation (Kla)-related- post-translational modifications (PTMs) regulating cell death in myocardial ischemia/reperfusion injury (MI/RI). Lactate elevates MICU3 levels by promoting H3K18la modification in the MICU3 promoter region, concurrently enhancing its interaction with AASR1. Upregulated MICU3 binds to VDAC1 to promote mitochondrial autophagy in MI/RI. Furthermore, MICU3 knockout increases pyruvate dehydrogenase (PDH) phosphorylation levels, thereby attenuating MI/RI. This suggests a potential link between Kla and phosphorylation in regulating mitochondrial autophagy. Accumulated lactate in MI/RI enhances the expression of the m6A reader protein YTHDF2 by promoting Kla, which in turn boosts G3BP1 expression and ultimately attenuates apoptosis. This reveals a potential connection between Kla and m6A in regulating apoptosis during MI/RI. The lactylase modifier KAT5 promotes LATS2 protein ubiquitination and degradation by acetylating STUB1, thereby inhibiting NLRP3-mediated pyroptosis to mitigate MI/RI. This suggests a regulatory role for Kla in pyroptosis via acetylation and ubiquitination pathways. Dexamethasone (Dex) suppresses iron death in MI/RI by upregulating NR3C1 phosphorylation, thereby inhibiting PDK4 expression and reducing MDH2 Kla levels. This reveals a mechanism whereby phosphorylation and Kla jointly regulate iron death.

In summary, in MI/RI, Kla transcends its role as a mere metabolic origin to function as a pivotal regulatory node, deeply integrated into the decision-making network governing cell death. Through modifying histones or signaling molecules, it engages in intricate cross-talk with other PTMs, such as phosphorylation, acetylation, m6A RNA methylation, and ubiquitination. This enables it to synergistically or antagonistically regulate autophagy, apoptosis, pyroptosis, and ferroptosis (Fig. 2). Consequently, future research focusing on how the Kla-PTMs network integrates diverse death signals and whether Kla mediates connections between various forms of cell death holds significant importance.

### Potential therapeutic targets for Kla in MI/RI

Kla is crucial for cellular metabolism, signal transmission, and gene expression. Particular molecules, including the lactate transporters MCT1 and MCT4, as well as essential enzymes related to lactacidification (*e.g.*, HDAC1–3), are pivotal to this process. Thus, treatments aimed at these compounds may possess potential therapeutic value for MI/RI.

### Targeting LDH

LDHA is the rate-limiting enzyme that governs lactate synthesis. During stress situations such as MI/RI, the heart experiences metabolic reprogramming marked by diminished aerobic oxidation and a substantial increase in glycolysis, resulting in a relative or absolute upregulation of LDHA activity. This leads to significant intracellular lactate buildup, supplying sufficient substrate for the Kla of proteins. Prior research has shown the therapeutic potential of targeting LDHA to regulate Kla in MI/RI. Research indicates elevated ACSL4 Kla levels in MI/RI, whereas LDHA deletion diminishes ACSL4 Kla, thereby reducing ferroptosis and easing MI/RI.^124^ Moreover, LDH expression is elevated in MI/RI, facilitating pyroptosis by augmenting NLRP3 K245la and intensifying MI/RI. LDHA knockdown diminishes infarct size and cardiac damage in I/R mice.^125^ FX11 and omartacil efficiently inhibit LDHA,^9^^,^^142^ presenting innovative therapeutic strategies for MI/RI. Consequently, at the onset of reperfusion or in individuals predisposed to severe metabolic disturbances, the administration of LDHA inhibitors (such as FX11^9^) may target the reduction of excessive lactate production at its origin, thereby obstructing the pathological modifications of lactate induced by its accumulation.

### Targeting MCTs

MCTs modulate lactate alterations by dynamically controlling intracellular lactate levels, thereby serving a crucial intermediary function in disease advancement. Studies demonstrate that in HF models, MCT4 expression on cardiomyocyte membranes is elevated as a compensatory mechanism to enhance lactate export and mitigate acidosis. This adaptive reaction may cause excessive intracellular lactate depletion, leading to reduced Kla at the K1897 site of the essential contractile protein α-MHC. This alteration is crucial for preserving the α-MHC-myosin interaction, and its absence directly undermines sarcomere architecture and contractile efficacy. Currently, pharmacological or genetic suppression of MCT4 increases intracellular lactate concentrations, reinstating α-MHC K1897 Kla and thereby enhancing cardiac function.^56^ This indicates that targeting MCT4 to maintain particular protective lactate alterations constitutes a feasible treatment approach in HF.

The increase in MCT4 expression in MI/RI is a significant factor in myocardial damage. It intensifies cellular metabolic distress and mortality by interfering with pyruvate metabolism and facilitating the cellular outflow of pyruvate as lactate. In contrast, the administration of the small-molecule medication (VB124) enhanced cellular metabolic state and mitigated oxidative stress and calcium overload, thereby increasing myocardial preservation and cardiac performance.^143^ MCT1 demonstrates significant expression in cardiac tissue. Post I/R, its expression exhibits a swift, temporary increase. Expression notably increased 15 min after reperfusion but was no longer significantly higher 60 min post-reperfusion. The administration of the competitive MCT1 inhibitor (d-lactate) before ischemia or during reperfusion resulted in increased infarct size and worse cardiac function. This suggests that the inhibition of MCT1 function intensifies MI/RI.^144^ It is worth noting that MCT1 can also serve as an effective route for drug delivery. Studies have shown that lactic acid anhydride in MI/RI can enhance myocardial cell uptake efficiency through MCT1, achieving significant cardioprotective effects at a lower dose.^145^ Therefore, enhancing or protecting the function of MCT1 may be a potential therapeutic strategy in MI/RI. For example, AZD-3965 is an inhibitor targeting the lactate transporter MCT1.^146^^,^^147^ Interfering with the activity of MCT1 may provide potential therapeutic benefits for MI/RI.

### Other strategies for targeting Kla

HDAC1–3 significantly impact the pathophysiology of MI/RI by modulating the acetylation and Kla modifications of genes. Studies suggest that many histone deacetylase inhibitors exhibit therapeutic promise in this domain. The US FDA-approved medicine saquinavir (SAHA) has been shown to augment cellular autophagy, facilitating myocardial cell survival during reperfusion stress, diminishing infarct size and enhancing heart function.^148^ Furthermore, it safeguards mitochondrial activity and diminishes the generation of ROS during ischemia.^149^ Enzalutamide (MS-275), another inhibitor, particularly inhibits HDAC1 activity in cardiac cell mitochondria, thereby reducing detrimental ROS levels after reperfusion and alleviating tissue damage.^150^ In addition to synthetic drugs, some natural compounds provide protective benefits through the modulation of deacetylases. Paeonol,^151^ resveratrol,^152^ SRT2104,^153^ and nicotinamide riboside^154^^,^^155^ have been shown to activate SIRT1–3, thereby exerting effects. Paeoniflorin has demonstrated significant efficacy in the treatment of MI/RI. Sodium lactate concurrently exhibits distinct therapeutic effects in the treatment of MI by increasing blood lactate levels.^56^^,^^155^ While certain targeted compounds are still in preclinical phases, they collectively demonstrate considerable therapeutic promise, indicating future possibilities as conventional targeted therapies. These systematic findings offer explicit and significant directional suggestions for future research.

### Clinical significance and prospect

The discovery of Histone Kla reveals an important connection between epigenetic control and cellular metabolism. Notably, lactate concentration, which is essential for metabolic functions, closely controls the levels of histone Kla. Lactate generation is influenced by several important metabolic processes, such as glutamine metabolism and glycolysis. Lactate levels are controlled by enzymes and transport proteins such as GLUTs, LDHA, and MCT, which in turn affect histone Kla levels. Histone acetyltransferases (HATs) CBP/p300 are the main enzymes that catalyze histone Kla, and HDAC1–3 and SIRT1–3 aid in its removal. It is yet unknown which variables affect the specificity and selectivity of HATs and HDACs. While some medications can control the course of disease by altering histone Kla levels, their exact mechanisms of action are yet unknown. The development of particular therapeutic options is complicated by the fact that many putative targets that modulate histone Kla also influence other PTMs. Therefore, the development of medications that precisely modulate histone Kla and the identification of exact targets are essential for future therapeutic approaches.

Kla forms cross-talk with PTMs such as phosphorylation, acetylation, m6A methylation, and ubiquitination in MI/RI and cooperatively or antagonistically regulates various cell death programs, including autophagy, apoptosis, pyroptosis, and ferroptosis. Moreover, Kla is also present in other forms of ischemic heart disease. For instance, during the acute MI phase, metabolic reprogramming leads to a surge in lactate production, while impaired clearance causes intracellular lactate accumulation. This lactate metabolic disorder impairs cardiac function through mechanisms such as disrupting ion homeostasis and affecting electrophysiological properties. Kla exhibits duality in this process: on one hand, histone Kla activates the expression of repair genes such as Lrg1 and Vegf-a, exerting anti-inflammatory and pro-angiogenic effects to promote tissue repair; on the other hand, specific modifications like Snail1 Kla drive the progression of pathological fibrosis. This functional diversity suggests that the ultimate effects of Kla depend on its specific molecular targets, cell types, and variations in lactate concentration across different pathological stages.

Exercise intervention studies offer essential insights into the dose–response relationship of lactate-induced alterations. Research demonstrates that swimming training markedly decreases lactate levels in mouse cardiac tissue, concomitantly downregulating the quantity of H3K18la modifications, thereby providing a protective effect against MI/RI.^121^ This indicates that exercise may affect Kla by modulating the concentration of its substrate, lactate. Intracellular lactate concentrations are known to range from 1 to 3 mmol/L at rest to over 15 mmol/L during exercise, establishing a potential concentration range for dose–response relationships.^19^ From an enzyme kinetics standpoint, the Km values for Kla by H3K18la 'erasers' like HDAC1–3 are in the micromolar (μM) range.^14^ Consequently, the observed drop in Kla levels after swimming training is mostly due to reduced synthesis rates resulting from lower lactate substrate availability, rather than changes in HDAC catalytic efficiency. Notably, this study revealed that four weeks of swimming training, while lowering lactate levels, also downregulated expression of the lactate-activated ‘writer’ EP300 and the ‘eraser’ HDAC3.^121^ This apparently paradoxical phenomenon illustrates the organism's meticulous reconfiguration of epigenetic regulatory networks while establishing a new metabolic homeostasis: under persistent limitations on lactate availability, cells sustain a steady-state equilibrium of Kla at reduced levels by collaboratively downregulating the expression of both EP300 and HDAC3.

Given that research on histone Kla is still in its infancy and there are still many open scientific concerns, we suggest the following study directions: (i) How much lactate concentration is necessary to cause Kla, and how does this threshold change depending on the tissue and cell type? (ii) What methods may be used to separate the distinct effects of Kla given the dynamic interactions and possible crosstalk between Kla and other PTMs? (iii) Apart from lactyl-CoA, LGSH, and adenosine lactate, are there other substrates involved in Kla? (iv) Apart from the enzymes found in previous studies, more investigation is required to find other enzymes linked to Kla and de-Kla. (v) Little is known about the effects of lactate level variations over time, the severity of myocardial ischemia, and the time to reperfusion. To provide novel information and strategies for the prevention and treatment of MI/RI, future research should concentrate on the function of lactation changes during ischemia and reperfusion as well as their connections to other pathogenic pathways. To advance the clinical use of histone Kla, a better understanding of its function and regulatory mechanisms in physiological and pathological processes—particularly in situations that largely rely on lactate and glycolysis—is necessary.

In summary, increased lactate production during MI/RI reflects the shift from aerobic metabolism to anaerobic glycolysis in cardiomyocytes. Lactate-induced Kla chemotaxis has a dual role in cardiac disease, either exacerbating injury or potentially facilitating repair. Kla is a PTM that interacts with acetylation, methylation, and phosphorylation modifications. Furthermore, as a newly discovered epigenetic alteration, Kla contributes to a complex regulatory network that underlies the pathophysiological mechanisms of MI/RI by playing a crucial regulatory role in several types of programmed cell death, such as autophagy, apoptosis, and pyroptosis. Key compounds that target lactate metabolism, including LDHA, MCTs, and HDACs, have shown potential to enhance MI/RI by modulating lactate production in preclinical experiments. Future comprehensive investigations into the precise targets and regulatory mechanisms of lactate modification in MI/RI, alongside the development of specific regulators for these pivotal molecules (such as MCT1/MCT4 inhibitors or agonists, LDHA inhibitors, etc.), will unveil novel perspectives and strategies for pharmacological intervention in MI/RI, ultimately furnishing a robust theoretical foundation and transformative pathways for mitigating MI/RI and enhancing patient prognosis.

## CRediT authorship contribution statement

**Xiaoting Yang:** Conceptualization. **Hui Wu:** Resources. **Di Liu:** Data curation. **Gang Zhou:** Data curation. **Dong Zhang:** Data curation. **Yanfang Liu:** Software. **Yi Li:** Software. **Tian Zhou:** Visualization, Conceptualization. **Yan Xiong:** Conceptualization.

## Funding

This work was supported by the National Natural Science Foundation of China (No. 82170260).

## Conflict of interests

The authors declare no competing interests.
